# Brain morphological alterations and their correlation to tumor differentiation and duration in patients with lung cancer after platinum chemotherapy

**DOI:** 10.3389/fonc.2022.903249

**Published:** 2022-08-04

**Authors:** Pin Lv, Guolin Ma, Wenqian Chen, Renyuan Liu, Xiaoyan Xin, Jiaming Lu, Shu Su, Ming Li, ShangWen Yang, Yiming Ma, Ping Rong, Ningyu Dong, Qian Chen, Xin Zhang, Xiaowei Han, Bing Zhang

**Affiliations:** ^1^ Department of Radiology, The Affiliated Drum Tower Hospital of Nanjing University Medical School, Nanjing, China; ^2^ Department of Radiology, China-Japan Friendship Hospital, Beijing, China; ^3^ The Comprehensive Cancer Centre of Drum Tower Hospital, Medical School of Nanjing University and Clinical Cancer Institute of Nanjing University, Nanjing, China; ^4^ Institute of Medical Imaging and Artificial Intelligence, Nanjing University, Nanjing, China; ^5^ Jiangsu Key Laboratory of Molecular Medicine, Nanjing, China; ^6^ Institute of Brain Science, Nanjing University, Nanjing, China

**Keywords:** chemotherapy-related brain impairment, lung cancers, platinum chemotherapy, three-dimensional T1-weighted imaging, differentiation degree of the tumor

## Abstract

**Objective:**

Chemotherapy-related brain impairments and changes can occur in patients with lung cancer after platinum chemotherapy and have a substantial impact on survivors’ quality of life. Therefore, it is necessary to understand the brain neuropathological alterations and response mechanisms to provide a theoretical basis for rehabilitation strategies. This study aimed to investigate the related brain morphological changes and clarified their correlation with clinical and pathological indicators in patients with lung cancer after platinum chemotherapy.

**Methods:**

Overall, 28 patients with chemotherapy, 56 patients without chemotherapy, and 41 healthy controls were categorized in three groups, matched for age, sex, and years of education, and included in the cross-sectional comparison of brain volume and cortical thickness. 14 matched patients before and after chemotherapy were subjected to paired comparison for longitudinal observation of brain morphological changes. Three-dimensional T_1_-weighted images were acquired from all participants, and quantitative parameters were calculated using the formula of the change from baseline. Correlation analysis was performed to evaluate the relationship between abnormal morphological indices and clinical information of patients.

**Results:**

Brain regions with volume differences among the three groups were mainly distributed in frontal lobe and limbic cortex. Additionally, significant differences in cerebrospinal fluid were observed in most ventricles, and the main brain regions with cortical thickness differences were the gyrus rectus and medial frontal cortex of the frontal lobe, transverse temporal gyrus of the temporal lobe, insular cortex, anterior insula, and posterior insula of the insular cortex. According to the paired comparison, decreased brain volumes in the patients after chemotherapy appeared in some regions of the frontal, parietal, temporal, and occipital lobes; limbic cortex; insular cortex; and lobules VI-X and decreased cortical thickness in the patients after chemotherapy was found in the frontal, temporal, limbic, and insular cortexes. In the correlation analysis, only the differentiation degree of the tumor and duration after chemotherapy were significantly correlated with imaging indices in the abnormal brain regions.

**Conclusions:**

Our findings illustrate the platinum-related brain reactivity morphological alterations which provide more insights into the neuropathological mechanisms of patients with lung cancer after platinum chemotherapy and empirical support for the details of brain injury related to cancer and chemotherapy.

## 1 Introduction

Chemotherapy-related brain impairment and cognitive abnormalities are frequent consequences in patients with lung malignancies after chemotherapy, especially with platinum drugs (1). A growing number of studies have shown that lung cancer survivors are at risk for cognitive dysfunction, which is usually characterized by various mental and/or psychological disorders, particularly affecting working memory, attention, and executive function (2). Chemotherapy-related brain changes may accelerate brain morphological changes; however, empirical evidence supporting this theory is limited (3, 4). Accordingly, it is urgent to understand the neurological morphology that can provide a theoretical basis for rehabilitation treatment strategies in survivors (5).

Over the past 20 years, research on the neuro-mechanism of platinum-based chemotherapy in patients with lung cancer has increased (6–8). In addition, some studies have explored the biological mechanism of the cognitive effects of chemotherapy drugs in small animals (9, 10). Neuroimaging plays an important role in neuropathology mechanistic research (11, 12), and with the development of magnetic resonance imaging (MRI) techniques, the detection of related morphological changes has become more convenient and contributed to understand some of chemotherapy-related cognitive impairment (CRCI) (13–15). An increasing number of neuroimaging studies have shown that the pathological cognitive symptoms of patients are particularly related to the altered structure of gray matter (GM) and abnormal brain volume, although the relationship between brain aging in patients with CRCI and biomarkers of neuroimaging is still only the tip of the iceberg (16). There is growing evidence that decreases in either the cortical surface area or thickness of the non-central nervous system cancers were found in multiple brain regions of interest, primarily within the frontal and temporal lobes (16, 17). Cortical thinning is a recognized imaging biomarker for brain injury in patients with breast cancer (18). Some studies have shown that the cortex gradually thins from 1 month to 1 year in patients with breast cancer after chemotherapy, and cortical thickness is positively correlated with language learning ability. Additionally, patients with chemotherapy have a greater reduction in temporal lobe volume than those without, and this was associated with a decrease in oral reading recognition scores (14, 19, 20). Hippocampal deformation or volume reduction is another abnormal change in patients receiving chemotherapy, and these changes are related to memory, long education years, poor self-reported cognitive function evaluation, and even a significant increase in inflammatory immune specific interleukin-6 and tumor necrosis factor-α (21–24). A recent study using gray matter density (GMD) as a parameter indicated that GMD decreased in the left inferior frontal gyrus, right middle frontal gyrus, right fusiform area, and both cerebellums in patients after chemotherapy. The number of chemotherapy cycles is negatively correlated with the general cognitive performance of patients (25). Although there are many confounding factors that cause brain dysfunction in clinical patients after chemotherapy, the application of neuroimaging technology makes it possible to noninvasively understand the neuropathological mechanism related to malignancies and chemotherapy and provide potential imaging biomarkers for early detection or prediction of brain injuries (26).

At present, brain structural data from magnetic resonance three-dimensional T_1_-weighted imaging (3D-T_1_WI) are mostly used to evaluate patients with brain trauma or Alzheimer’s disease-related cognitive impairment but have not been applied in malignancy-associated morphological changes with or without chemotherapy (27, 28). In this study, we hypothesized that patients with lung cancer would exhibit abnormal volume and cortical thickness in different brain regions after platinum chemotherapy, and that these morphological changes would be related to the clinical information of the patients. Our study aimed to verify this hypothesis with volume and cortical thickness indexes based on high-resolution 3D-T_1_WI. The association between altered morphology indexes and clinical data of patients was also evaluated.

## 2 Methods

### 2.1 Study population

This study was approved by Medical Ethics Committee of the Nanjing Drum Tower Hospital. The patients/participants and written informed consent was obtained from each participant. All participants were retrospectively identified at our hospital between January 2019 and December 2020. The inclusion criteria were as follows: 1) all patients were first diagnosed with cancer without metastasis; 2) patients diagnosed with lung cancer based on the final pathological results who had a chemotherapy; 3) patients with at least one high-resolution 3D-T_1_WI was performed before or after chemotherapy; 4) patients with no other brain and psychiatric diseases and no history of psychotropic drug use; 5) patients without obvious cerebral atrophy and a Fazekas score of chronic ischemic hypoxia<grade II after evaluation by two radiologists with >5 years of work experience; and 6) right-handed patients. The exclusion criteria were as follows: 1) patients with a duration after chemotherapy of<30 days; 2) patients with non-platinum chemotherapy; 3) patients without 3D-T_1_WI; and 4) patients with brain metastasis, stroke, arterial aneurysm, cerebral hemorrhage, or use of psychotropic drugs.

**Figure 1:**
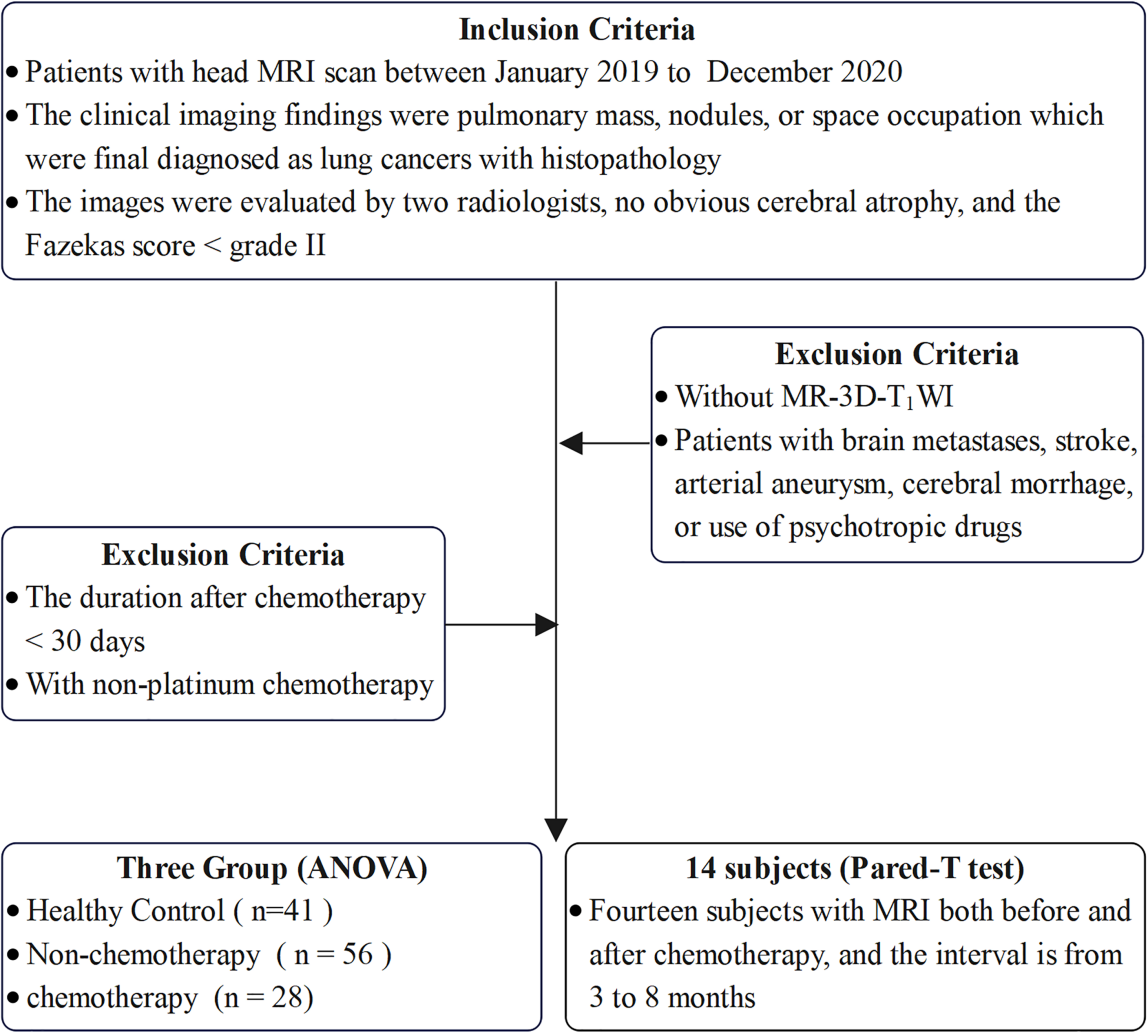
Flow diagram of the inclusion criteria for the study population.

We conducted a cross-sectional observation of the brain volume and cortical thickness between three groups, including 28 patients with chemotherapy, 56 patients without chemotherapy, and 41 healthy controls (HC), in which the three groups were matched for age, sex, and years of education. The inclusion criteria for HC were: 1) absence of brand and systemic diseases and neurological symptoms and signs; 2) absence of psychiatric diseases and history of psychotropic drug use; 3) normal head MRI and 3D-T_1_WI findings; 4) absence of obvious cerebral atrophy and a Fazekas score of chronic ischemic hypoxia<grade II after evaluation by two radiologists with >5 years of working experience; and 5) right-handed patients. In addition, for the longitudinal observation of brain morphological changes, we compared 14 paired patients before and after chemotherapy. The inclusion criteria are summarized in **Figure 1**.

### 2.2 Image acquisition

Imaging data were acquired using a 3.0T MRI scanner (Ingenia CX, Philips) with a dStream head 32-channel coil. The 3D-T_1_WI images were acquired using three-dimensional fast-spoiled gradient-echo sequences. The parameters were set as follows: repetition time/echo time, 6.6/3.0 ms; flip angle, 8°; field of view, 250 × 250 × 180 mm^3^; matrix size, 250 × 250 × 180; voxel size, 1.00 × 1.00 × 1.00 mm^3^ with no gap; and number of signal average, 1.

### 2.3 Image processing and morphological analysis

**Figure 2:**
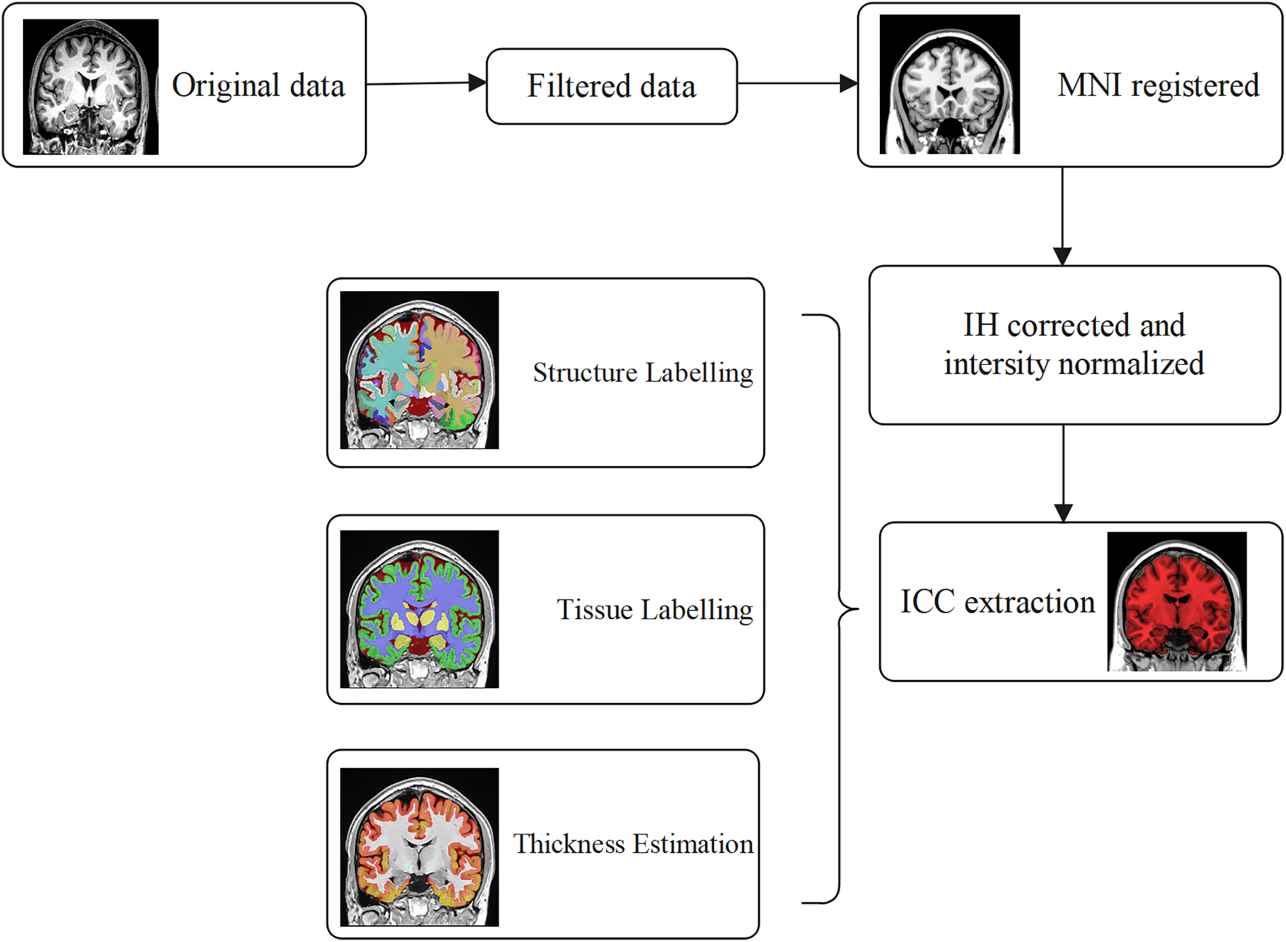
The preprocessing of 3D-T_1_WI data with online software vol2Brain. Nonlocal noise reduction filter, nonuniformity correction, MNI spatial registration, intensity normalization, and ICC extraction were included. The results of global tissue estimation (GM, WM, and CSF) following the segmentation results of macro structure and subcortical structure outputs automatically.

The 3D-T_1_WI data in NIFTI format were analyzed using the online software vol2Brain (https://www.volbrain.upv.es), which divides the human brain into >100 subregions to automatically calculate their volume and cortical thickness (29). The preprocessing process includes a nonlocal noise reduction filter, nonuniformity correction, MNI spatial registration, intensity normalization, and intracranial cavity extraction. Then, the volume results of global tissues, GM, white matter, and cerebrospinal fluid (CSF) and the results of macro and subcortical structures were calculated. The whole brain was substantially segmented into the frontal, parietal, temporal, and occipital lobes, limbic cortex, insular cortex, CSF, and cerebellar vermis. Each region was separately divided into several subregions in which the volume and cortical thickness (except for the CSF and cerebellar vermis) could be calculated (total, right, and left), the data processing flow is shown in **Figure 2**.

**Figure 3:**
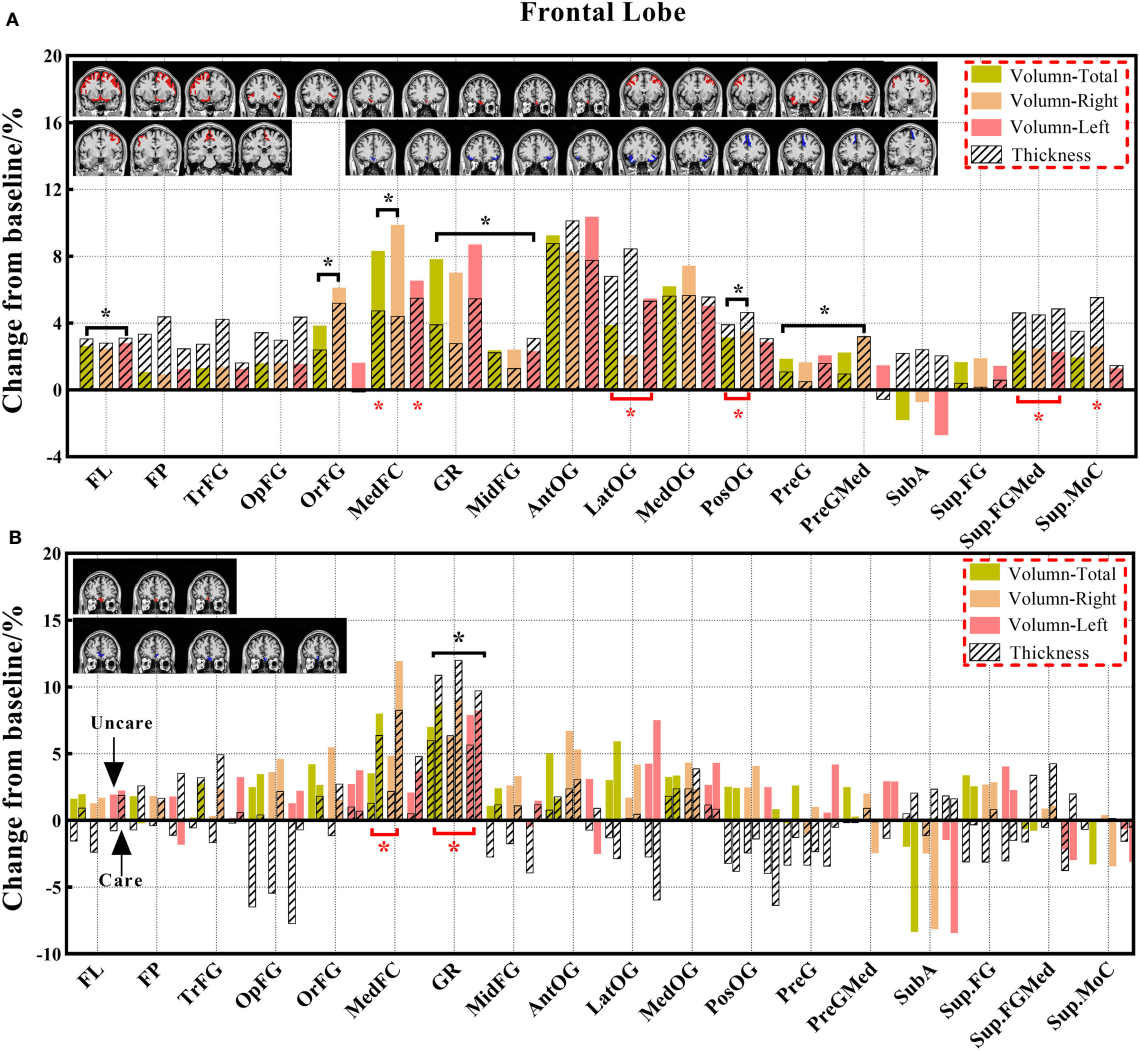
The changes in brain volume and cortical thickness from baseline in the frontal lobe. **(A)** represents the results of 14 subjects and **(B)** shows the results of the three groups. Solid and shaded histograms represent volume and cortical thickness, respectively. The coronal with red is the significant difference subregion for volume, and the blue for thickness. Green, orange, and pink on behalf of total, right and left. Black* and red* indicate a statistically significant difference (P<0.05) in volume and cortical thickness, respectively (The labels appearing in this figure and their interpretation are the same as those in the **Figures 4**–**9**).

**Figure 4:**
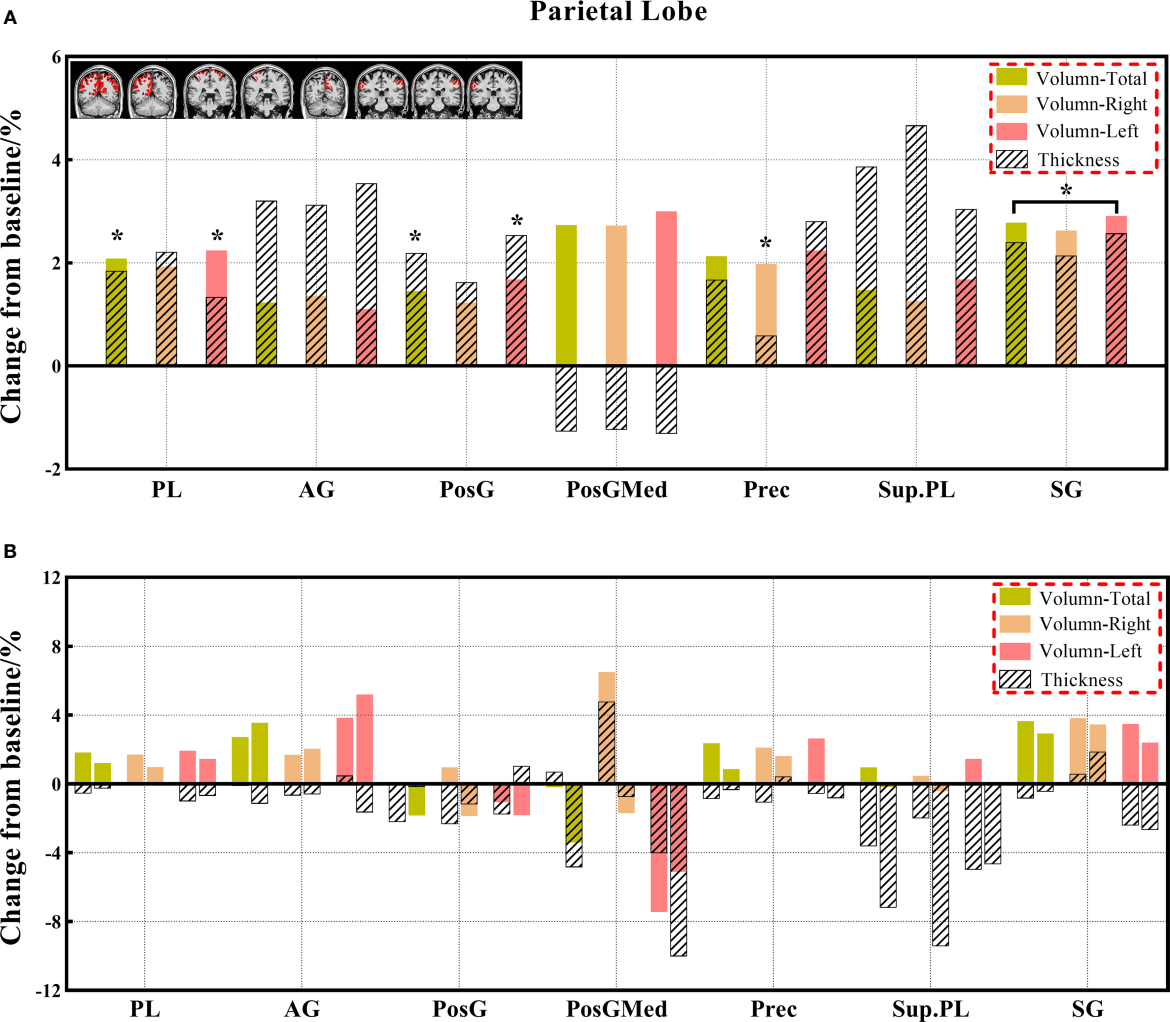
**(A, B)** The changes in brain volume and cortical thickness from baseline in the parietal lobe. Black * and red* indicate a statistically significant difference (P<0.05) in volume and cortical thickness.

**Figure 5:**
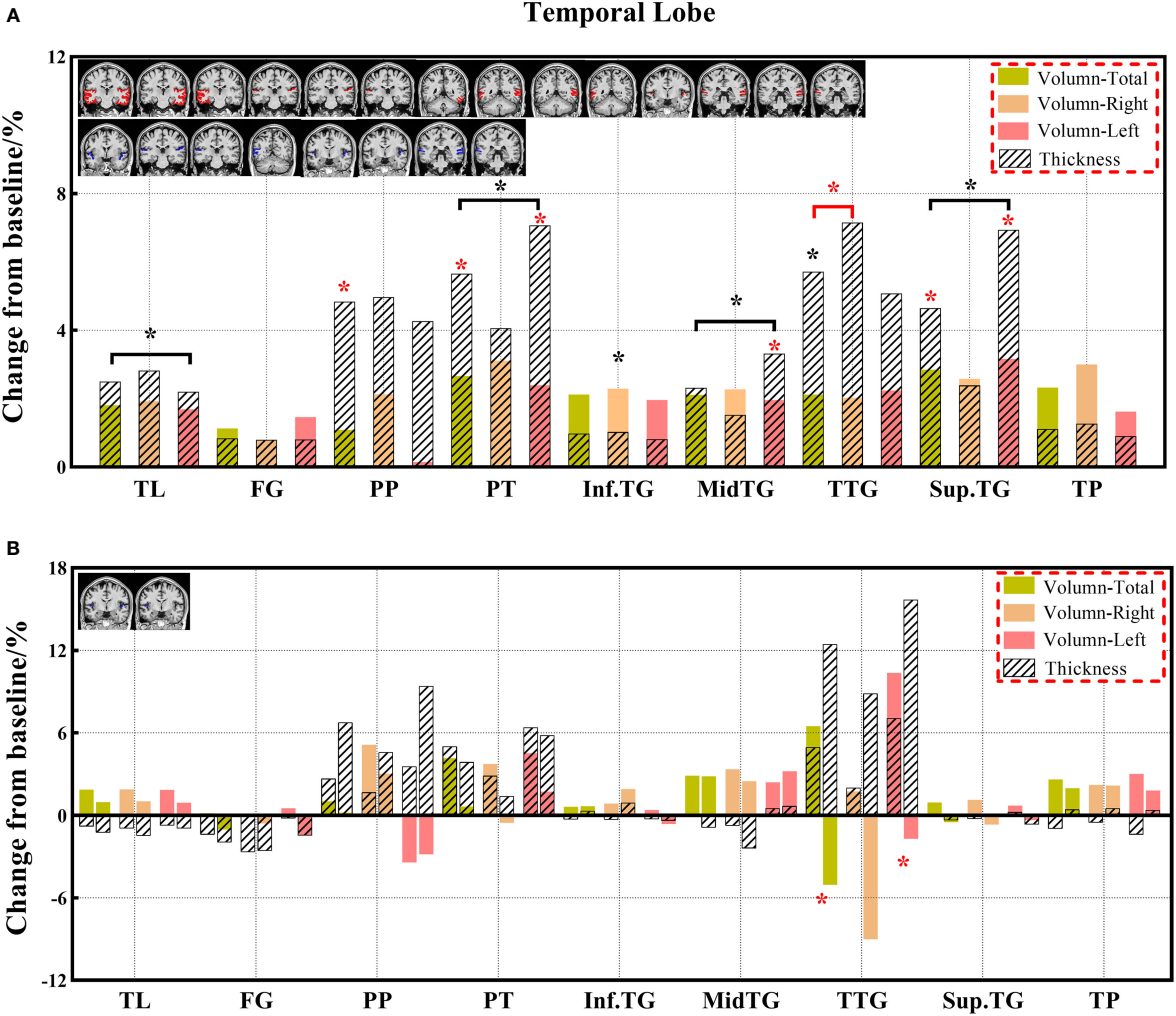
**(A, B)** The changes in brain volume and cortical thickness from baseline in the temporal lobe. Black * and red* indicate a statistically significant difference (P<0.05) in volume and cortical thickness.

**Figure 6:**
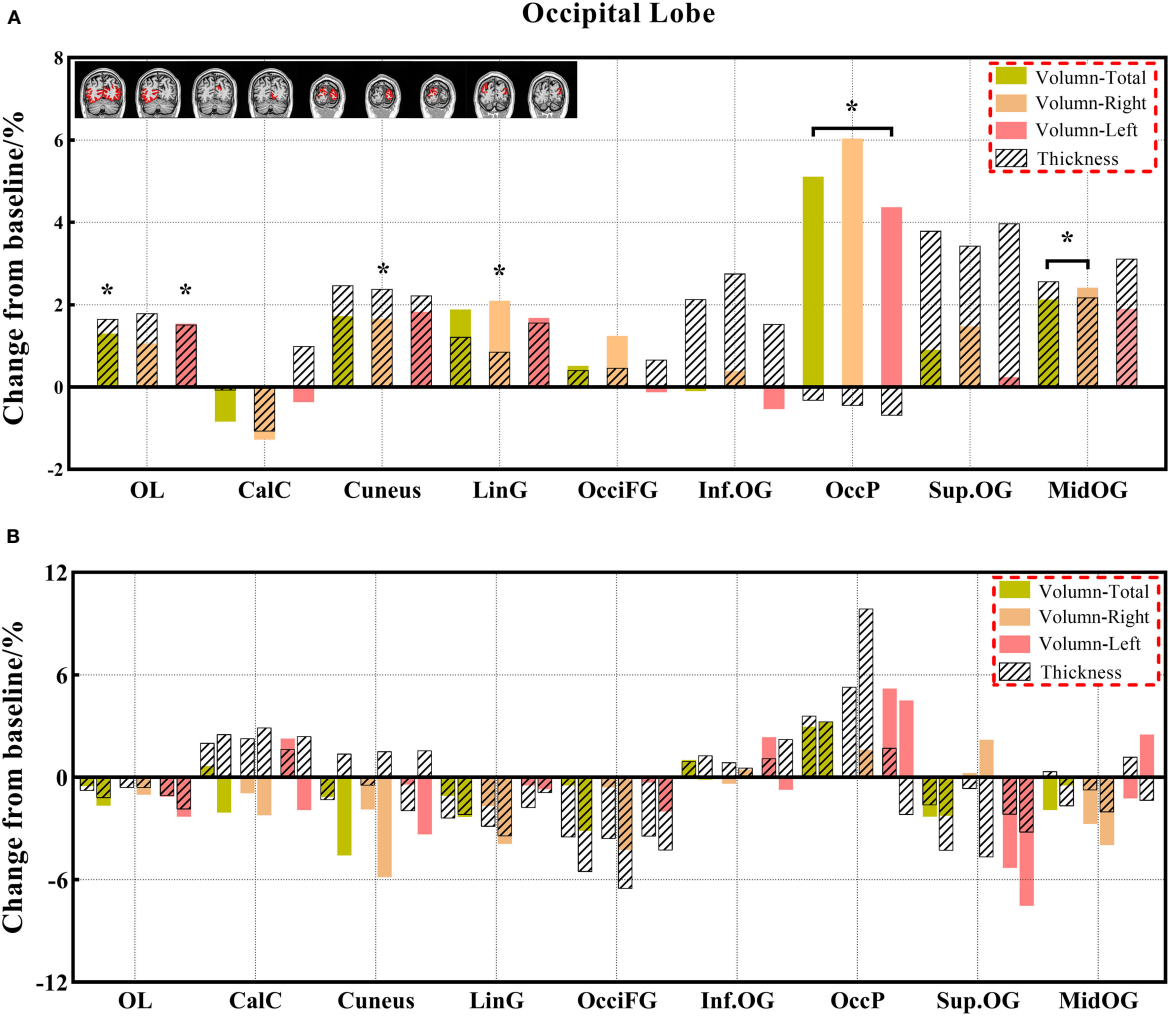
**(A, B)** The changes in brain volume and cortical thickness from baseline in the occipital lobe. Black * and red* indicate a statistically significant difference (P<0.05) in volume and cortical thickness.

**Figure 7:**
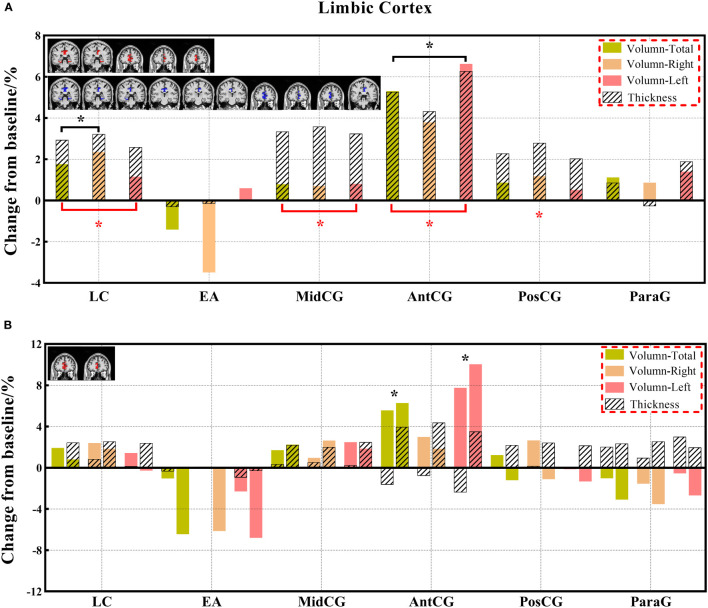
**(A, B)** The changes in brain volume and cortical thickness from baseline in the limbic cortex. Black * and red* indicate a statistically significant difference (P<0.05) in volume and cortical thickness.

**Figure 8:**
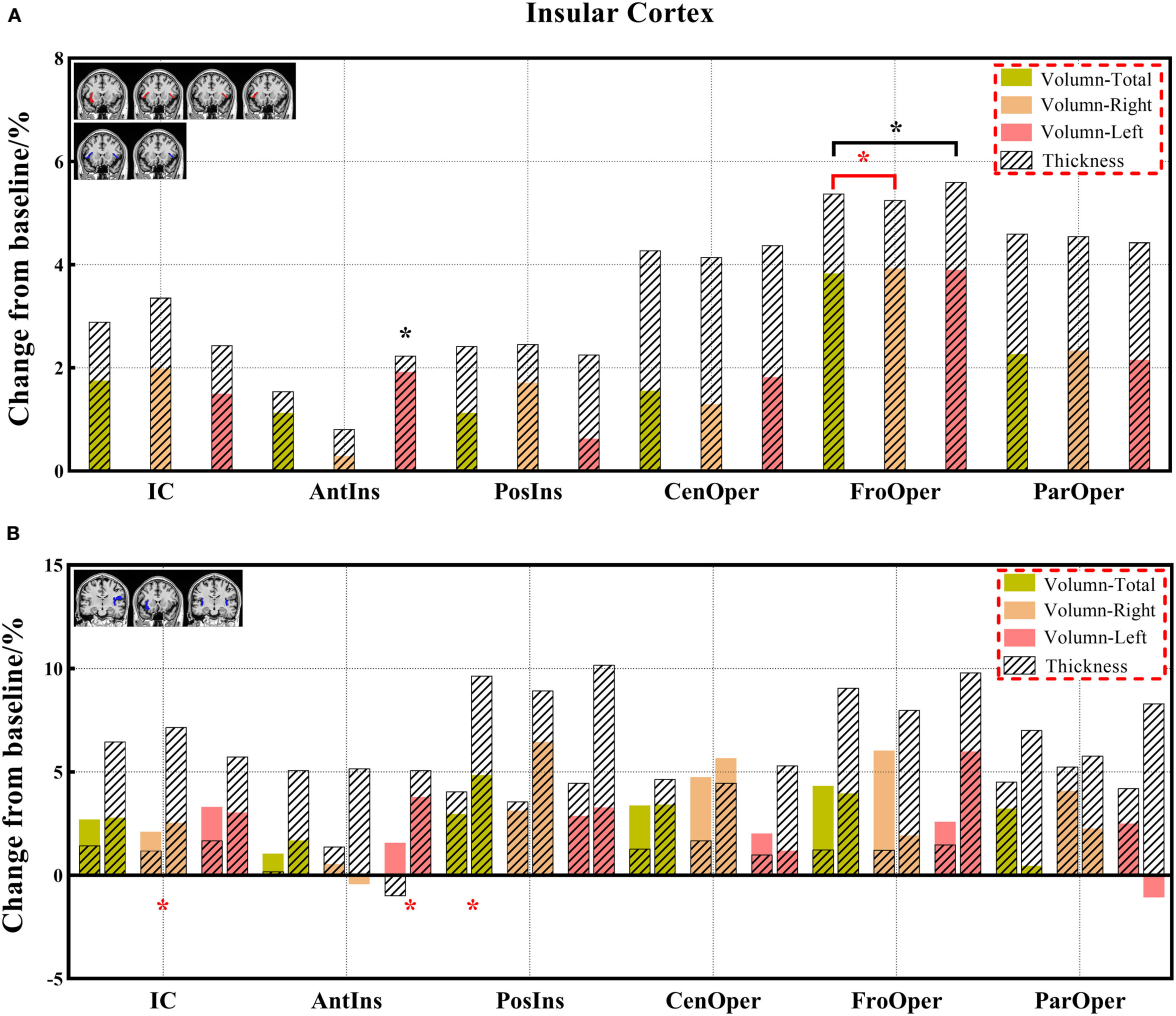
**(A, B)** The changes in brain volume and cortical thickness from baseline in the insular cortex. Black * and red* indicate a statistically significant difference (P<0.05) in volume and cortical thickness.

**Figure 9:**
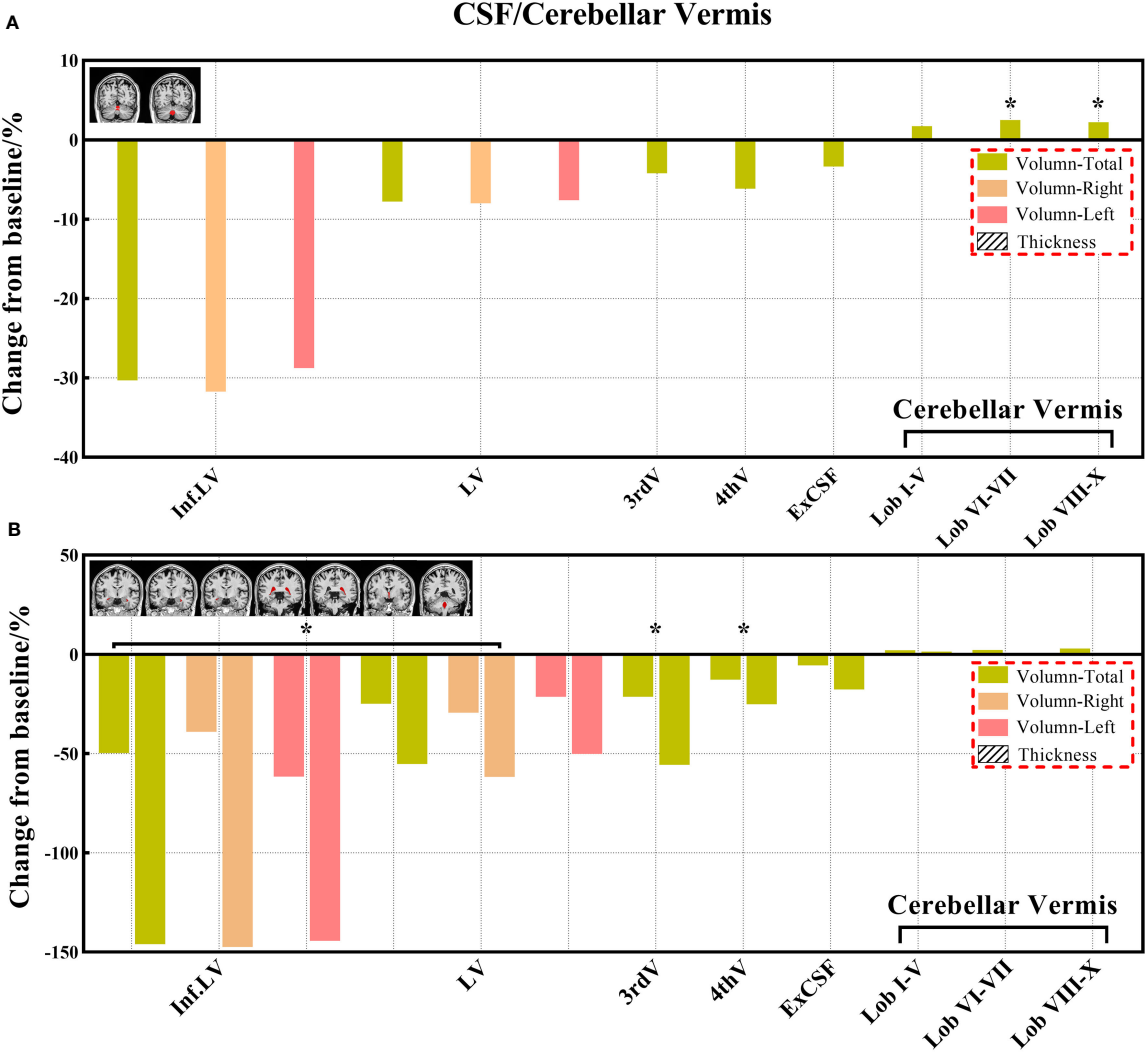
**(A, B)** The changes of volume from baseline in the CSF and cerebellar vermis. (No cortical thickness data for the CSF and cerebellar vermis). Black * and red * indicate a statistically significant difference (P<0.05) in volume and cortical thickness.

Variations in the brain volume and cortical thickness were quantified by calculating the rate of change for a better evaluation of brain morphological changes in this study (**Figures 3**–**9**). The quantitative parameters were calculated according to the following formula:


$$Change\;from\;baseline\;\left(\%\right)=\frac{A-B}{A}\times 100$$

*where A represents the data before chemotherapy for the 14 paired patients or HC, and B is the data after chemotherapy for the 14 patients after chemotherapy in longitudinal analysis or patients with/without chemotherapy in the cross-section analysis*.

### 2.4 Statistical analysis

The demographic and clinical data of the participants were analyzed using SPSS (version 26, IBM Corp.) and Gretna package software (https://www.nitrc.org/projects/gretna/). A one-way analysis of covariance was used to compare differences in the volume and cortical thickness between the three groups with age, sex, and years of education as covariates. Then, a two-sample t-test was performed between every two groups to identify the source of differences. The paired t-test was performed in the 14 paired patients to evaluate the volume and cortical thickness changes before and after chemotherapy. Correlation analysis was mainly used to evaluate the relationship between abnormal imaging indices and clinical information (the tumor size, tumor stage, and degree of differentiation), and it was performed only for brain regions with significant differences between the groups. Statistical significance was set at P< 0.05.

## 3 Results

### 3.1 Clinical data

The results indicated no significant differences in sex, age, and years of education between the three groups (all, P > 0.05). The average number of chemotherapy cycles was 10.53 ± 8.93 and 6.00 ± 1.41 for the chemotherapy and paired groups, respectively. The average duration after chemotherapy was 793.60 ± 693.65 and 221 ± 59.4 days for the chemotherapy and paired groups, respectively. The main pathological types of cancer were squamous cell carcinoma and adenocarcinoma in both patient groups with and without chemotherapy. The demographic and clinical data are shown in **Table 1**, while the abbreviations of brain regions are shown in **Supplemental Table 1**.

**Table 1:** Demographic and clinical data.

Contents		Three groups			P-value	Two paired groups
		Healthy control	Chemotherapy	Non-chemotherapy		
Gender (male/female)		22/19	20/8	37/19	0.28	11/3
Age (mean ± SD)		58.83 ± 8.21	60.54 ± 7.48	58.8 ± 8.11	0.61	60.36 ± 6.62
Education (years, mean ± SD)		9.10 ± 4.86	9.00 ± 4.43	9.13 ± 3.45	0.99	8.21 ± 3.77
Histological types (cases/proportion)	Squamous cell carcinoma	–	12 (42.86)	12 (21.43)	–	5(50)
	Adenocarcinoma	–	13 (46.43)	34 (60.71)	–	5(35.71)
	Others	–	3 (10.71)	10 (17.86)	–	2(14.29)
Chemotherapy cycle (cycles, mean ± SD)		–	10.53 ± 8.93	–	–	6.00 ± 1.41
Duration after chemotherapy (days, mean ± SD)		–	793.60 ± 693.65	–	–	221.00 ± 59.4

### 3.2 Cross-sectional analysis of the three groups

#### 3.2.1 Comparison of volume indexes

The main brain regions with volume differences between the three groups were the gyrus rectus (total, right, and left) of the frontal lobe and anterior cingulate gyrus (total and left) of the limbic cortex. In addition, a significant difference in the subregions of CSF occurred in the inferior lateral ventricle (total, right, and left), lateral ventricle (total and right), and third and fourth ventricles (**Supplemental Table 2**).

Compared with the HC group, the chemotherapy group showed significantly decreased volumes in the gyrus rectus (total, right, and left) and medial frontal cortex (right) of the frontal lobe, and anterior cingulate gyrus (left) of the limbic cortex, except for the increased volume of the entorhinal area (total). The CSF volume was significantly increased in all brain chambers in the chemotherapy group. Compared with the HC group, the non-chemotherapy group showed significantly increased volumes in the gyrus rectus (total, right, and left) of the frontal lobe, anterior cingulate gyrus (total and left) of the limbic cortex, and transverse temporal gyrus (left) of the temporal lobe. The CSF volume was significantly increased in the inferior lateral ventricle (total and left), lateral ventricle (right), and third ventricle in the non-chemotherapy group. Compared to the non-chemotherapy group, the chemotherapy group showed significantly increased volumes in the third ventricle (**Supplemental Tables 3**–**5**).

#### 3.2.2 Comparison of cortical thickness indexes

The main brain regions with cortical thickness differences between the three groups were the gyrus rectus (total, right, and left) and medial frontal cortex (total and right) of the frontal lobe; transverse temporal gyrus (total and left) of the temporal lobe; and insular cortex (right), anterior insula (left), and posterior insula (total) of the insular cortex. The mean cortical thickness was ranked from high to low as follows: HC, non-chemotherapy, and chemotherapy groups (**Supplemental Table 6**).

Compared with the HC group, the chemotherapy group showed a significantly decreased cortical thickness in the gyrus rectus (total, right, and left), medial frontal cortex (total and right), and posterior orbital gyrus (left) of the frontal lobe; transverse temporal gyrus (total and left) of the temporal lobe; occipital fusiform gyrus (total and right) of the occipital lobe; and insular cortex(total and right), posterior insula (total, right, and left), and frontal operculum (total, left) of the insular cortex. Compared with the HC group, the non-chemotherapy group showed a significantly decreased cortical thickness in the gyrus rectus (total, right, and left) of the frontal lobe and an increased cortical thickness in the opercular inferior frontal gyrus (total and left) of frontal lobe. Compared to the non-chemotherapy group, the chemotherapy group showed a significantly decreased cortical thickness in the medial frontal cortex (right) of the frontal lobe and anterior insula (total and left) and frontal operculum (total) of the insular cortex (**Supplemental Tables 7**–**9**).

### 3.3 Analysis of the paired groups

#### 3.3.1 Comparison of volume indexes

Decreased brain volumes in the patients after chemotherapy appeared in the frontal lobe (total, right, and left), gyrus rectus (total, right, and left), orbital inferior frontal gyrus (total and right), medial frontal cortex (total and right), middle frontal gyrus (total, right, and left), posterior orbital gyrus (total and right), precentral gyrus (total, right, and left), and precentral gyrus medial segment (total and right) of the frontal lobe; parietal lobe (total and left), postcentral gyrus (total and left), precuneus (right), and supramarginal gyrus (total, right, and left) of the parietal lobe; temporal lobe (total, right, and left), planum temporale (total, right, and left), inferior temporal gyrus (right), middle temporal gyrus (total, right, and left), superior temporal gyrus (total, right, and left), and transverse temporal gyrus (total) of the temporal lobe; occipital lobe (total and left), cuneus (right), lingual gyrus (right), middle occipital gyrus (total and right), and occipital pole (total, right, and left) of the occipital lobe; limbic cortex (total and right), anterior cingulate gyrus (total, right, and left) of the limbic cortex; anterior insula (left), frontal operculum (total, right, and left) of the insular cortex; and lobules VI-VII, lobules VIII-X. There was no statistically significant difference in the CSF volume in both patient groups before and after chemotherapy; however, the CSF volume increased after chemotherapy based on the calculation of its change from baseline (**Supplemental Table 10**).

#### 3.3.2 Comparison of cortical thickness indexes

Decreased cortical thickness in patients after chemotherapy was observed in the medial frontal cortex (total and left), lateral orbital gyrus (total, right, and left), posterior orbital gyrus (total and right), superior frontal gyrus medial segment (total, right, and left), and superior motor cortex (right) of the frontal lobe; planum polare (total), planum temporale (total and left), middle temporal gyrus (left), superior temporal gyrus (total and left), and transverse temporal gyrus (total and right) of the temporal lobe; limbic cortex (total, right, and left), anterior cingulate gyrus (total, right, and left), middle cingulate gyrus (total, right, and left), and posterior cingulate gyrus (right) of the limbic cortex; and frontal operculum (total, right) of the insular cortex (**Supplemental Table 11**).

### 3.4 Correlation analysis

#### 3.4.1 Three groups

For brain regions with significant differences between the chemotherapy and HC groups, the cortical thickness in the medial frontal cortex (total and right), insular cortex (total), posterior insula (total and right), and frontal operculum (total and left) showed a positive correlation with the degree of tumor differentiation degree (P< 0.05). The CSF volume of the third ventricle showed a negative correlation with the differentiation degree of the tumor when analyzed between the chemotherapy and HC groups and the chemotherapy and non-chemotherapy groups (both, P = 0.01).

For the brain regions with significant differences between the chemotherapy and non-chemotherapy groups, the cortical thickness in the medial frontal cortex (right) of the frontal lobe, anterior insula (total), and frontal operculum (total and left) of the insular lobe showed a positive correlation with the degree of tumor differentiation degree (P< 0.05) (**Supplemental Table 12**).

#### 3.4.2 Paired groups

Significant differences in brain volumes were positively correlated with the duration after chemotherapy (P< 0.05) in the precentral gyrus medial segment (total and right) of the frontal lobe, postcentral gyrus (total) of the parietal lobe, and occipital pole (total, right, and left) of the occipital lobe. Significant differences in brain volumes were positively correlated with the degree of tumor differentiation degree (P< 0.05) in the planum temporale (total and left) of the temporal lobe.

Significant differences in cortical thickness were negatively correlated with the duration after chemotherapy (P< 0.05) in the medial frontal cortex (total), posterior orbital gyrus (total and right), and superior frontal gyrus medial segment (total and right) of the frontal lobe; planum polare (total), middle temporal gyrus (left), and superior temporal gyrus (total and left) of the temporal lobe; anterior cingulate gyrus (total and right) of the limbic cortex; and frontal operculum (total, right) of the insular cortex. The significant difference in cortical thickness was positively correlated with the degree of tumor differentiation degree (P< 0.05) in the medial frontal cortex (left) and lateral orbital gyrus (total, right, and left) of the frontal lobe and superior temporal gyrus (left) of the temporal lobe. However, the significant difference in cortical thickness was negatively correlated with the degree of tumor differentiation degree (P< 0.05) in the superior motor cortex (right) of the frontal lobe (**Supplemental Table 13**).

## 4 Discussion

Cancer survivors account for an increasing proportion of patients. It is important to understand the neuropathological mechanism and neuroanatomical basis of alterations related to chemotherapy (11, 18). Therefore, in this study, we identified early abnormal volumes and cortical thicknesses in different brain regions and altered CSF volumes in different brain ventricular spaces in patients with lung cancer with or without chemotherapy using 3D-T_1_WI. Most results supported the decrease in brain volume in both the observational comparison results of the three groups and the matched comparison results of the two groups. The patients with or without chemotherapy showed a significant volume decrease, mainly in the frontal lobe and limbic cortex, and an extensive increase in CSF volumes in the ventricles in the analysis of the three groups, whereas the volumes decreased in the analysis of the paired groups. Moreover, patients with or without chemotherapy showed a significant decrease in cortical thickness, mainly in the frontal lobe, temporal lobe, and insular lobe in the analysis of the three groups and a more extensive decrease in brain regions in the analysis of the paired groups. Most of these altered regions are related to cognitive, executive, emotional, and motor functions (5, 6, 30).

Results of the analysis in the three groups showed significant differences in brain regions in the gyrus rectus of the frontal lobe and anterior cingulate gyrus of the limbic cortex. Furthermore, when compared with the HC group, the chemotherapy group also showed a significant decrease in the volume of the medial frontal cortex and entorhinal area, and the non-chemotherapy group showed a significant decrease in the volume of the transverse temporal gyrus. These findings demonstrate novelty that chemotherapy may accelerate volume reduction in relevant brain regions (18, 31, 32). However, the long-term stability of this effect after chemotherapy is unclear (33, 34). The analysis of the paired groups showed that decreased volumes were found in more extensive regions, including the frontal, parietal, temporal, occipital, limbic, insular, and partial cerebellar regions. This result is consistent with that of previous studies (18, 35–37) and is probably caused by the shorter duration after chemotherapy in the patients of the paired groups, in which the brain regions were temporarily injured and in a recovering state partly (21, 38).

Notably, a significant increase in CSF was found in the chemotherapy and non-chemotherapy groups based on the results of the analysis of the three groups. In the paired groups, the amount of CSF tended to increase after chemotherapy according to the change from baseline, although there were no statistical differences between these two groups. Previous studies have shown that patients with cancer but without chemotherapy have abnormal brain changes as a possible mechanism of tumor-related endocrine dysfunction and pro-inflammatory immune response (39, 40). The decreased volume of brain regions and increased CSF volume can be used as potential neuroimaging markers of chemotherapy-related brain injury at an early stage because of their high sensitivity to pharmacological toxicity (41, 42). These factors are important findings for brain idiosyncratic morphological alterations. However, the mechanism of chemotherapy-related brain injury is still controversial. Some studies have shown that chemotherapy drug are neurotoxic. The drugs would induce after-treatment changes to overall brain volume in both white and gray matter, and the severity of impairment increasing with the dose increase. On the other hand, tumors also effect the central nervous system, damage the integrity of the blood-brain barrier. neuroinflammatory responses including increased pro-inflammatory cytokines and reduced anti-inflammatory cytokines which result in the increases of reactive oxidative stress and mitochondrial dysfunction (43, 44). Macroscopically, cognitive function was impaired and brain morphology changed. The quantitative evaluation of cognitive function and the neurobiological mechanism aspects after chemotherapy still need to be further studied.

In this study, we identified differentiated cortical thicknesses in the frontal, temporal, and insular regions between the three groups. Furthermore, the cortical thickness in the frontal, temporal, occipital, and insular lobes also showed a significant decrease in the chemotherapy group compared to the HC group, and the cortical thickness of the frontal and insular lobes was significantly lower in the chemotherapy group than in the non-chemotherapy group. The statistically significant reduction in cortical thickness mainly occurs in the frontal and temporal lobes of patients after chemotherapy (18). These results are consistent with those of previous studies (18, 24, 45, 46). Results of the analysis of the paired groups showed that decreased volumes were found in more brain regions, including the limbic cortex and other regions of the frontal, temporal, and insular cortexes. These results are consistent with those in previous studies demonstrating the prediction of brain morphological abnormality in patients with malignancies after chemotherapy (18, 47). The change in cortical thickness indicates that there will be eventual accompanying clinical manifestations related to different degrees of brain alterations. Prominently, cortical thickness has become a reliable morphological indicator (32, 48).

Few studies have reported correlations between neuroimaging morphological indicators and clinical data in patients undergoing chemotherapy (49). In the present study, the cortical thickness of the medial frontal cortex and insular regions showed positive correlations, and the CSF volume of the third ventricle was negatively correlated with the degree of tumor differentiation in the chemotherapy group. It may be that the worse the tumor differentiation and the longer the average chemotherapy cycles, the more serious the damage to the medial prefrontal lobe and insular regions, and the greater the amount of CSF in the third ventricle (50, 51). This situation was slightly different in the paired groups, mainly because the volumes of multiple brain regions distributed in the frontal, parietal, and occipital lobes were positively correlated with the duration after chemotherapy, except that the volume of the planum temporale was positively correlated with the differentiation degree of the tumor in the paired groups. This result is different from that of another study (52). It is possible that short chemotherapy cycles may have caused edema in some regions in this study. In addition, cortical thickness in the frontal, temporal, limbic, and insular lobes was negatively correlated with the duration of chemotherapy. The longer the duration after chemotherapy, the more serious the damage to these related regions (23, 31, 32, 36). The cortical thickness of regions in the frontal and temporal lobes positively correlated with the degree of differentiation of the tumor, except the superior motor cortex in the frontal lobe showed a negative correlation. The reason for the negative correlation of the superior motor cortex is unclear, but is possibly due to its compensation (25, 53).

Interestingly, we did not find a correlation between morphological changes in the brain and tumor size, clinical stage, or chemotherapy cycles. During the analysis process, we did not perform a multiple regression model; therefore, the results of this study may also reflect unknown multivariable or mediating effects. Additionally, the sample size of patients after chemotherapy was still small, which may have resulted in a certain degree of bias, although we used the data of the paired groups. We did not use the cognitive correlation scale score or correlation analysis with the patient’s blood-related metabolite indicators in this retrospective analysis. Finally, we could not provide internal information about the potential biological mechanism of brain morphological changes based on neuroimaging due to the lack of animal and biological experiments. Future neuroimaging studies should consider adding biomarkers and contributing additional neurobiological insights into cancer-related brain injury.

## 5 Conclusion

In conclusion, this study revealed specific brain morphological changes based on neuroimaging in patients with lung cancer with or without chemotherapy, and the main manifestations were increased CSF volume, decreased cortical thickness and cerebral parenchyma atrophy; furthermore, it clarified the correlation between altered brain morphological indexes and tumor differentiation and the duration after treatment. Our findings provide more insights into the neural mechanisms of patients with lung cancer after platinum chemotherapy and empirical support for brain morphological injury related to cancer and chemotherapy.

## Data availability statement

The raw data supporting the conclusions of this article will be made available by the authors, without undue reservation.

## Ethics statement

The studies involving human participants were reviewed and approved by Medical Ethics Committee of the Nanjing Drum Tower Hospital. The patients/participants provided their written informed consent to participate in this study.

## Author contributions

XH and BZ designed, organized, and revised this work. PL and GM contributed to data processing and drafting the original manuscript. WC, RL, PR, ML, SY, YM, and ND were responsible for MR scanning. SS provided clinical and pathological information. XX and XZ evaluated all radiological images, calculated, and reviewed relevant indicators. PL, WC, JL, and QC performed the statistical analyses. All authors contributed to the article and approved the submitted version.

## Funding

This work was supported by the National Natural Science Foundation of China (82171908 XH, 81720108022 BZ, 81971596, XZ, 82001793, JL), the Fundamental Research Funds for the Central Universities, Nanjing University (2020-021414380462), the Key Scientific Research Project of Jiangsu Health Committee (K2019025), Industry and Information Technology Department of Nanjing (SE179-2021), Educational Research Project of Nanjing Medical University (2019ZC036), and the Project of Nanjing Health Science and Technology Development (YKK19055). The funders had no role in the study design, data collection and analysis, decision to publish, or preparation of the manuscript.

## Conflict of interest

The authors declare that the research was conducted in the absence of any commercial or financial relationships that could be construed as a potential conflict of interest.

The reviewer YZ declared a shared parent affiliation with the authors PL, WC, RL, XX, JL, SS, ML, SY, YM, PR, ND, QC, XZ, XH, and BZ to the handling editor at the time of review.

## Publisher’s note

All claims expressed in this article are solely those of the authors and do not necessarily represent those of their affiliated organizations, or those of the publisher, the editors and the reviewers. Any product that may be evaluated in this article, or claim that may be made by its manufacturer, is not guaranteed or endorsed by the publisher.
