# Supplementary material for: Brain morphological alterations and their correlation to tumor differentiation and duration in patients with lung cancer after platinum chemotherapy
Source: Front Oncol. 2022 Aug 4;12:903249. doi: 10.3389/fonc.2022.903249 (PMC9396961; doi:10.3389/fonc.2022.903249)
Supplement: Supplementary file 1 [file Table_1.docx]

**Supplementary Table 1** Brain divisions and Abbreviation

| Lobe | SubRegion | Abbreviation | Lobe | SubRegion | Abbreviation |
| --- | --- | --- | --- | --- | --- |
| Frontal Lobe  (FL) | Frontal pole | FP | Occipital lobe  (OL) | Calcarine cortex | CalC |
|  | Triangular inf. frontal gyrus | TrFG |  | Cuneus | Cuneus |
|  | Opercular inf. frontal gyrus | OpFG |  | Lingual gyrus | LinG |
|  | Orbital inf. frontal gyrus | OrFG |  | Occipital fusiform gyrus | OcciFG |
|  | Medial frontal cortex | MedFC |  | Inf. occipital gyrus | Inf.OG |
|  | Gyrus rectus | GR |  | Occipital pole | OccP |
|  | Middle frontal gyrus | MidFG |  | Sup. occipital gyrus | Sup.OG |
|  | Anterior orbital gyrus | AntOG |  | Middle occipital gyrus | MidOG |
|  | Lateral orbital gyrus | LatOG | Limbic cortex (LC) | Entorhinal area | EA |
|  | Medial orbital gyrus | MedOG |  | Middle cingulate gyrus | MidCG |
|  | Posterior orbital gyrus | PosOG |  | Anterior cingulate gyrus | AntCG |
|  | Precentral gyrus | PreG |  | Posterior cingulate gyrus | PosCG |
|  | Precentral gyrus medial segment | PreGMed |  | Parahippocampal gyrus | ParaG |
|  | Subcallosal area | SubA | Insular cortex  (IC) | Anterior insula | AntIns |
|  | Sup. frontal gyrus | Sup.FG |  | Posterior insula | PosIns |
|  | Sup. frontal gyrus medial segment | Sup.FGMed |  | Central operculum | CenOper |
|  | Sup. motor cortex | Sup.MoC |  | Frontal operculum | FroOper |
| Parietal Lobe  (PL) | Angular gyrus | AG |  | Parietal operculum | ParOper |
|  | Postcentral gyrus | PosG | CSF | Inf. Lateral Ventricle | Inf.LV |
|  | Postcentral gyrus medial segment | PosGMed |  | Lateral Ventricle | LV |
|  | Precuneus | Prec |  | 3rd Ventricle | 3rdV |
|  | Sup. parietal lobule | Sup.PL |  | 4th Ventricle | 4thV |
|  | Supramarginal gyrus | SG |  | External CSF | ExCSF |
| Temporal Lobe  (TL) | Fusiform gyrus | FG | Cerebellar vermis | Lobules I-V | Lob I-V |
|  | Planum polare | PP |  | Lobules VI-VII | Lob VI-VII |
|  | Planum temporale | PT |  | Lobules VIII-X | Lob VIII-X |
|  | Inf. temporal gyrus | Inf.TG |  |  |  |
|  | Middle temporal gyrus | MidTG |  |  |  |
|  | Transverse temporal gyrus | TTG |  |  |  |
|  | Sup. temporal gyrus | Sup.TG |  |  |  |
|  | Temporal pole | TP |  |  |  |

**Supplementary Table 2** Brain volume differences among three groups with ANOVA

|  | **Region** | **NC(mm^3^)** | **Uncare** | **Care** | **F** | **P** |
| --- | --- | --- | --- | --- | --- | --- |
| Frontal Lobe | GR-T | 3.77 | 3.51 | 3.45 | 4.54 | 0.013 |
|  | GR-R | 1.97 | 1.85 | 1.80 | 3.87 | 0.024 |
|  | GR-L | 1.80 | 1.66 | 1.65 | 3.90 | 0.023 |
| Limbic Cortex | AntCG-T | 10.67 | 10.08 | 10.00 | 3.28 | 0.041 |
|  | AntCG-L | 5.74 | 5.29 | 5.16 | 5.56 | 0.005 |
| CSF | Inf.LV-T | 0.53 | 0.79 | 1.32 | 4.29 | 0.016 |
|  | Inf.LV-R | 0.27 | 0.38 | 0.68 | 3.97 | 0.021 |
|  | Inf.LV-L | 0.26 | 0.41 | 0.64 | 4.36 | 0.015 |
|  | LV-T | 19.18 | 23.96 | 29.99 | 4.08 | 0.019 |
|  | LV-R | 8.56 | 11.08 | 13.92 | 5.23 | 0.007 |
|  | 3rd | 1.23 | 1.49 | 1.88 | 7.79 | 0.001 |
|  | 4th | 1.53 | 1.73 | 1.93 | 4.29 | 0.016 |

*Uncare: non-chemotherapy; Care: chemotherapy

**Supplementary Table 3** Brain volume differences between chemotherapy and HC group

|  |  | **Region** | **NC (mm^3^)** | **Care** | **T** | **P** |  | **Region** | **NC** | **Care** | **T** | **P** |
| --- | --- | --- | --- | --- | --- | --- | --- | --- | --- | --- | --- | --- |
| Volume | Frontal Lobe | GR-T | 3.77 | 3.45 | 2.38 | 0.020 | CSF | Inf.LV-T | 0.53 | 1.32 | -2.44 | 0.017 |
|  |  | GR-R | 1.97 | 1.80 | 2.29 | 0.025 |  | Inf.LV-R | 0.27 | 0.68 | -2.20 | 0.032 |
|  |  | GR-L | 1.80 | 1.65 | 2.09 | 0.041 |  | Inf.LV-L | 0.26 | 0.64 | -2.69 | 0.009 |
|  |  | MedFC-R | 1.49 | 1.32 | 2.21 | 0.031 |  | LV-T | 19.18 | 29.99 | -2.96 | 0.004 |
|  | Limbic Cortex | EA-T | 4.22 | 4.49 | -2.08 | 0.041 |  | LV-R | 8.56 | 13.92 | -3.36 | 0.001 |
|  |  | AntCG-L | 5.74 | 5.16 | 2.49 | 0.015 |  | LV-L | 10.62 | 16.07 | -2.55 | 0.013 |
|  |  |  |  |  |  |  |  | 3rd | 1.23 | 1.88 | -4.27 | 0.000 |
|  |  |  |  |  |  |  |  | 4th | 1.53 | 1.93 | -3.06 | 0.003 |
|  |  |  |  |  |  |  |  | ExCSF | 167.81 | 193.70 | -2.41 | 0.019 |

**Supplementary Table 4** Brain volume differences between non-chemotherapy and HC group

|  |  | **Region** | **NC (mm^3^)** | **UnCare** | **T** | **P** |
| --- | --- | --- | --- | --- | --- | --- |
| Volume | Frontal Lobe | GR-T | 3.77 | 3.51 | 2.61 | 0.011 |
|  |  | GR-R | 1.97 | 1.85 | 2.21 | 0.029 |
|  |  | GR-L | 1.80 | 1.66 | 2.63 | 0.010 |
|  | Limbic Cortex | AntCG-T | 10.67 | 10.08 | 2.36 | 0.020 |
|  |  | AntCG-L | 5.74 | 5.29 | 2.91 | 0.004 |
|  | Temporal Lobe | TTG-L | 1.58 | 1.42 | 2.26 | 0.026 |
|  | CSF | Inf.LV-T | 0.53 | 0.79 | -2.15 | 0.034 |
|  |  | Inf.LV-L | 0.26 | 0.41 | -2.21 | 0.030 |
|  |  | LV-R | 8.56 | 11.08 | -2.15 | 0.034 |
|  |  | 3rd | 1.23 | 1.49 | -2.18 | 0.032 |

**Supplementary Table 5** Brain volume differences between chemotherapy and non-chemotherapy group

|  |  | **Region** | **Uncare (mm^3^)** | **Care** | **T** | **P** |
| --- | --- | --- | --- | --- | --- | --- |
| Volume | CSF | 3rd | 1.49 | 1.88 | 2.01 | 0.047 |

**Supplementary Table 6** Cortical thickness differences among three groups with ANOVA

|  | **Region** | **NC(mm)** | **Nncare** | **Care** | **F** | **P** |
| --- | --- | --- | --- | --- | --- | --- |
| Frontal Lobe | MedFC-T | 3.16 | 3.12 | 2.96 | 3.17 | 0.045 |
|  | MedFC-R | 3.23 | 3.16 | 2.96 | 4.03 | 0.020 |
|  | GR-T | 3.22 | 3.03 | 2.87 | 8.18 | <0.000 |
|  | GR-R | 3.32 | 3.11 | 2.92 | 8.48 | <0.000 |
|  | GR-L | 3.11 | 2.93 | 2.80 | 5.73 | 0.004 |
| Temporal Lobe | TTG-T | 1.57 | 1.49 | 1.37 | 3.33 | 0.039 |
|  | TTG-L | 1.64 | 1.52 | 1.38 | 5.57 | 0.005 |
| Insular Cortex | IC-R | 2.63 | 2.60 | 2.44 | 3.08 | 0.050 |
|  | AntIns-L | 3.40 | 3.44 | 3.23 | 3.38 | 0.037 |
|  | PosIns-T | 2.92 | 2.80 | 2.64 | 3.23 | 0.043 |

**Supplementary Table 7** Cortical thickness differences between chemotherapy and HC group

|  |  | **Region** | **NC (mm)** | **Care** | **T** | **P** |  | | **Region** | **NC** | | **Care** | | **T** | | **P** | |  |
| --- | --- | --- | --- | --- | --- | --- | --- | --- | --- | --- | --- | --- | --- | --- | --- | --- | --- | --- |
| Thickness | Frontal Lobe | GR-T | 3.22 | 2.87 | 3.82 | 0.000 | Occipital Lobe | | OcciFG-T | 2.33 | | 2.45 | | -2.03 | | 0.046 | |  |
|  |  | GR-R | 3.32 | 2.92 | 4.15 | 0.000 |  |  | OcciFG-R | 2.42 | | 2.58 | | -2.11 | | 0.039 | |  |
|  |  | GR-L | 3.11 | 2.80 | 3.02 | 0.004 | Insular Cortex | IC-T | | 2.67 | | 2.50 | | 2.02 | | 0.047 | |  |
|  |  | MedFC-T | 3.16 | 2.96 | 2.25 | 0.028 |  | IC-R | | | 2.63 | | 2.44 | | 2.19 | | 0.032 | |
|  |  | MedFC-R | 3.23 | 2.96 | 2.64 | 0.010 |  | PosIns-T | | | 2.92 | | 2.64 | | 2.32 | | 0.023 | |
|  |  | PosOG-L | 2.89 | 3.08 | -2.03 | 0.047 |  | PosIns-R | | | 3.02 | | 2.75 | | 2.13 | | 0.037 | |
|  | Temporal Lobe | TTG-T | 1.57 | 1.37 | 2.40 | 0.019 |  | PosIns-L | | | 2.81 | | 2.52 | | 2.25 | | 0.028 | |
|  |  | TTG-L | 1.64 | 1.38 | 3.04 | 0.003 |  | FroOper-T | | | 2.36 | | 2.15 | | 2.24 | | 0.029 | |
|  |  |  |  |  |  |  |  | FroOper-L | | | 2.36 | | 2.13 | | 2.16 | | 0.035 | |

**Supplementary Table 8** Cortical thickness differences between non-chemotherapy and HC group

|  |  | **Region** | **NC (mm)** | **UnCare** | **T** | **P** |
| --- | --- | --- | --- | --- | --- | --- |
| Thickness | Frontal  Lobe | GR-T | 3.22 | 3.03 | 2.77 | 0.007 |
|  |  | GR-R | 3.32 | 3.11 | 2.62 | 0.010 |
|  |  | GR-L | 3.11 | 2.93 | 2.50 | 0.014 |
|  |  | OpFG-T | 1.72 | 1.83 | -2.11 | 0.037 |
|  |  | OpFG-L | 1.69 | 1.82 | -2.20 | 0.030 |

**Supplementary Table 9** Cortical thickness differences between non-chemotherapy and chemotherapy

|  |  | **Region** | **Uncare (mm)** | **Care** | **T** | **P** |
| --- | --- | --- | --- | --- | --- | --- |
| Thickness | Frontal Lobe | MedFC-R | 3.16 | 2.96 | 2.02 | 0.047 |
|  | Insular Cortex | IC-R | 2.60 | 2.44 | 2.09 | 0.039 |
|  |  | AntIns-T | 3.45 | 3.29 | 2.04 | 0.045 |
|  |  | AntIns-L | 3.44 | 3.23 | 2.54 | 0.013 |
|  |  | FroOper-T | 2.33 | 2.15 | 2.17 | 0.033 |

**Supplementary Table 10** Brain volume differences in the paired groups

|  | **Region** | **Pre(mm^3^)** | **Post** | **T** | **P** |  | **Region** | **Pre** | **Post** | **T** | **P** |
| --- | --- | --- | --- | --- | --- | --- | --- | --- | --- | --- | --- |
| Frontal Lobe | FL-T | 159.84 | 155.63 | 2.97 | 0.011 | Temporal Lobe | TL-T | 95.15 | 93.43 | 2.93 | 0.012 |
|  | FL-R | 79.79 | 77.82 | 2.97 | 0.011 |  | TL-R | 47.28 | 46.37 | 3.00 | 0.010 |
|  | FL-L | 80.04 | 77.81 | 2.91 | 0.012 |  | TL-L | 47.87 | 47.06 | 2.72 | 0.017 |
|  | GR-T | 3.65 | 3.36 | 3.07 | 0.009 |  | PT-T | 3.65 | 3.55 | 4.41 | <0.001 |
|  | GR-R | 1.91 | 1.78 | 2.55 | 0.024 |  | PT-R | 1.68 | 1.62 | 2.66 | 0.020 |
|  | GR-L | 1.73 | 1.58 | 3.55 | 0.004 |  | PT-L | 1.97 | 1.93 | 2.74 | 0.017 |
|  | OrFG-T | 2.08 | 2.00 | 3.24 | 0.006 |  | Inf.TG-R | 11.86 | 11.58 | 2.72 | 0.018 |
|  | OrFG-R | 1.07 | 1.01 | 3.89 | 0.002 |  | MidTG-T | 27.09 | 26.52 | 4.26 | 0.001 |
|  | MedFC-T | 2.66 | 2.44 | 2.75 | 0.017 |  | MidTG-R | 13.68 | 13.37 | 3.99 | 0.002 |
|  | MedFC-R | 1.41 | 1.27 | 3.04 | 0.010 |  | MidTG-L | 13.42 | 13.15 | 3.06 | 0.009 |
|  | MidFG-T | 35.43 | 34.58 | 3.61 | 0.003 |  | Sup.TG-T | 12.01 | 11.67 | 4.01 | <0.001 |
|  | MidFG-R | 17.66 | 17.24 | 3.14 | 0.008 |  | Sup.TG-R | 6.19 | 6.03 | 2.35 | 0.035 |
|  | MidFG-L | 17.76 | 17.35 | 3.55 | 0.004 |  | Sup.TG-L | 5.83 | 5.64 | 4.89 | 0.000 |
|  | PosOG-T | 5.30 | 5.14 | 3.25 | 0.006 |  | TTG-T | 2.87 | 2.81 | 2.23 | 0.044 |
|  | PosOG-R | 2.56 | 2.47 | 2.97 | 0.011 | Occipital Lobe | OL-T | 72.25 | 71.31 | 2.40 | 0.032 |
|  | PreG-T | 24.16 | 23.71 | 3.84 | 0.002 |  | OL-L | 35.92 | 35.36 | 2.77 | 0.016 |
|  | PreG-R | 11.78 | 11.58 | 2.49 | 0.027 |  | CuneusR | 4.09 | 4.02 | 2.35 | 0.035 |
|  | PreG-L | 12.38 | 12.13 | 4.57 | <0.001 |  | LinG-R | 8.75 | 8.56 | 2.16 | 0.050 |
|  | PreGMed-T | 5.07 | 4.96 | 2.72 | 0.018 |  | MidOG-T | 9.77 | 9.56 | 2.47 | 0.028 |
|  | PreGMed-R | 2.51 | 2.43 | 3.25 | 0.006 |  | MidOG-R | 4.59 | 4.48 | 2.28 | 0.040 |
| Parietal Lobe | PL-T | 104.02 | 101.85 | 2.57 | 0.023 |  | OccP-T | 4.59 | 4.35 | `3.62 | 0.003 |
|  | PL-L | 52.05 | 50.88 | 3.05 | 0.009 |  | OccP-R | 1.99 | 1.87 | 3.66 | 0.003 |
|  | PosG-T | 17.92 | 17.66 | 2.33 | 0.037 |  | OccP-L | 2.60 | 2.49 | 2.79 | 0.015 |
|  | PosG-L | 9.12 | 8.97 | 2.51 | 0.026 | Limbic Cortex | LC-T | 39.59 | 38.89 | 2.29 | 0.040 |
|  | Prec-R | 11.02 | 10.80 | 2.20 | 0.046 |  | LC-R | 20.24 | 19.76 | 2.44 | 0.030 |
|  | SG-T | 15.65 | 15.22 | 5.56 | 0.000 |  | AntCG-T | 10.11 | 9.57 | 4.31 | <0.001 |
|  | SG-R | 7.78 | 7.57 | 3.70 | 0.003 |  | AntCG-R | 4.79 | 4.61 | 2.60 | 0.022 |
|  | SG-L | 7.88 | 7.65 | 2.71 | 0.018 |  | AntCG-L | 5.32 | 4.97 | 3.32 | 0.005 |
| Insular Cortex | AntIns-L | 4.16 | 4.08 | 2.22 | 0.045 | Cerebellar Vermis | Lob VI-VII | 1.87 | 1.82 | 3.03 | 0.010 |
|  | FroOper-T | 3.32 | 3.19 | 4.17 | <0.001 |  | Lob VIII-X | 2.72 | 2.66 | 2.38 | 0.033 |
|  | FroOper-R | 1.67 | 1.61 | 3.11 | 0.008 |  |  |  |  |  |  |
|  | FroOper-L | 1.65 | 1.59 | 2.90 | 0.012 |  |  |  |  |  |  |

**Supplementary Table 11** Cortical thickness differences in the paired groups

|  | **Region** | **Pre(mm)** | **Post** | **T** | **P** |  | **Region** | **Pre** | **Post** | **T** | **P** |
| --- | --- | --- | --- | --- | --- | --- | --- | --- | --- | --- | --- |
| Frontal Lobe | MedFC-T | 3.07 | 2.93 | 2.42 | 0.031 | Limbic Cortex | LC-T | 3.19 | 3.10 | 3.58 | 0.003 |
|  | MedFC-L | 3.06 | 2.89 | 2.56 | 0.024 |  | LC-R | 3.17 | 3.07 | 3.36 | 0.005 |
|  | LatOG-T | 2.72 | 2.54 | 3.03 | 0.010 |  | LC-L | 3.21 | 3.13 | 2.90 | 0.012 |
|  | LatOG-R | 2.70 | 2.48 | 3.02 | 0.010 |  | AntCG-T | 3.60 | 3.41 | 3.74 | 0.002 |
|  | LatOG-L | 2.74 | 2.59 | 2.64 | 0.020 |  | AntCG-R | 3.59 | 3.43 | 3.47 | 0.004 |
|  | PosOG-T | 3.05 | 2.93 | 2.39 | 0.032 |  | AntCG-L | 3.61 | 3.39 | 3.69 | 0.003 |
|  | PosOG-R | 3.15 | 3.01 | 2.81 | 0.015 |  | MidCG-T | 2.83 | 2.73 | 2.74 | 0.017 |
|  | Sup.FGMed-T | 2.84 | 2.71 | 3.03 | 0.010 |  | MidCG-R | 2.88 | 2.78 | 2.98 | 0.011 |
|  | Sup.FGMed-R | 2.89 | 2.76 | 2.85 | 0.014 |  | MidCG-L | 2.76 | 2.67 | 2.22 | 0.045 |
|  | Sup.FGMed-L | 2.77 | 2.64 | 2.97 | 0.011 |  | PosCG-R | 3.20 | 3.11 | 2.68 | 0.019 |
|  | Sup.MoC-R | 2.61 | 2.47 | 2.60 | 0.022 | Insular Cortex | FroOper-T | 2.26 | 2.14 | 2.33 | 0.037 |
| Temporal Lobe | PP-T | 1.29 | 1.22 | 2.21 | 0.046 |  | FroOper-R | 2.29 | 2.17 | 2.20 | 0.047 |
|  | PT-T | 1.57 | 1.48 | 2.82 | 0.015 |  |  |  |  |  |  |
|  | PT-L | 1.64 | 1.52 | 2.98 | 0.011 |  |  |  |  |  |  |
|  | MidTG-L | 3.08 | 2.98 | 2.26 | 0.042 |  |  |  |  |  |  |
|  | Sup.TG-T | 2.26 | 2.15 | 2.71 | 0.018 |  |  |  |  |  |  |
|  | Sup.TG-L | 2.33 | 2.17 | 3.98 | 0.002 |  |  |  |  |  |  |
|  | TTG-T | 1.55 | 1.46 | 2.19 | 0.047 |  |  |  |  |  |  |
|  | TTG-R | 1.46 | 1.35 | 2.30 | 0.038 |  |  |  |  |  |  |

**Supplementary Table 12** Correlation analysis for the three groups

|  |  |  | Region | Parameter | r | P |
| --- | --- | --- | --- | --- | --- | --- |
| Care vs NC | Volume | CSF | 3rd | Differentiation degree | -0.478 | 0.01 |
|  | Thickness | Frontal Lobe | MedFC-T |  | 0.483 | 0.009 |
|  |  |  | MedFC-R |  | 0.483 | 0.009 |
|  |  | Insular Cortex | IC-T |  | 0.404 | 0.033 |
|  |  |  | PosIns-T |  | 0.38 | 0.046 |
|  |  |  | PosIns-R |  | 0.455 | 0.015 |
|  |  |  | FroOper-T |  | 0.51 | 0.006 |
|  |  |  | FroOper-L |  | 0.486 | 0.009 |
| Care vs Uncare | Volume | CSF | 3rd |  | -0.478 | 0.01 |
|  | Thickness | Frontal Lobe | MedFC-R |  | 0.483 | 0.009 |
|  |  | Insular Cortex | AntIns-T |  | 0.471 | 0.011 |
|  |  |  | FroOper-T |  | 0.51 | 0.006 |
|  |  |  | FroOper-L |  | 0.486 | 0.009 |

**Supplementary Table 13** Correlation analysis for the paired groups

|  |  | Region | Parameter | r | P |
| --- | --- | --- | --- | --- | --- |
| Volume | Frontal Lobe | PreGMed-T | Time | 0.573 | 0.032 |
|  |  | PreGMed-R |  | 0.558 | 0.038 |
|  | Parietal Lobe | PosG-T |  | 0.63 | 0.016 |
|  | Occipital lobe | OccP-T |  | 0.621 | 0.018 |
|  |  | OccP-R |  | 0.561 | 0.037 |
|  |  | OccP-L |  | 0.58 | 0.03 |
|  | Temporal Lobe | PT-T | Differentiation degree | 0.547 | 0.043 |
|  |  | PT-L |  | 0.584 | 0.028 |
| Thickness | Frontal Lobe | MedFC-T | Time | -0.661 | 0.01 |
|  |  | PosOG-T |  | -0.659 | 0.01 |
|  |  | PosOG-R |  | -0.751 | 0.02 |
|  |  | Sup.FGMed-T |  | -0.563 | 0.036 |
|  |  | Sup.FGMed-R |  | -0.545 | 0.044 |
|  | Temporal Lobe | PP-T |  | -0.605 | 0.022 |
|  |  | MidTG-L |  | -0.609 | 0.021 |
|  |  | Sup.TG-T |  | -0.632 | 0.015 |
|  |  | Sup.TG-L |  | -0.737 | 0.003 |
|  | Limbic Cortex | AntCG-T |  | -0.623 | 0.017 |
|  |  | AntCG-R |  | -0.667 | 0.009 |
|  | Insular Cortex | FroOper-T |  | -0.58 | 0.03 |
|  |  | FroOper-R |  | -0.602 | 0.023 |
|  | Frontal Lobe | MedFC-L | Differentiation degree | 0.637 | 0.014 |
|  |  | LatOG-T |  | 0.575 | 0.031 |
|  |  | LatOG-R |  | 0.593 | 0.026 |
|  |  | LatOG-L |  | 0.548 | 0.042 |
|  |  | Sup.MoC-R |  | -0.568 | 0.034 |
|  | Temporal Lobe | Sup.TG-L |  | 0.625 | 0.017 |
